# A Preliminary Study of Effect of Melatonin on Inflammation and Hypoxia‐Related Factors in a Mouse Model of Elastase‐Induced Intracranial Aneurysm

**DOI:** 10.1002/brb3.70371

**Published:** 2025-02-28

**Authors:** NarenYa, Yan Feng, Yongxing Su, Le Chen, Yan Liu, Zhongwu Sun, Zhengfei Ma

**Affiliations:** ^1^ Department of Neurology The First Affiliated Hospital of Anhui Medical University Hefei Anhui Province People's Republic of China; ^2^ Department of Neurology Suzhou Hospital of Anhui Medical University Suzhou Anhui Province People's Republic of China

**Keywords:** hypoxia, immune cells, intracranial aneurysm, melatonin

## Abstract

**Introduction:**

Intracranial aneurysms (IAs) are relatively common cerebrovascular anomalies. Melatonin could modulate inflammatory and offers neuroprotective effects, and its role in IA has not been fully elucidated.

**Methods:**

An elastase‐induced IA mouse model was constructed and melatonin (150 mg/kg) was administered to investigate its therapeutic effects on IA. Aneurysm formation was observed by bromophenol blue gelatin perfusion, and the pathology changes in IA mice were examined using hematoxylin‐eosin (HE) staining. The potential mechanisms of melatonin treatment of IA were explored using western blot, enzyme‐linked immunosorbent, real‐time qPCR, immunohistochemistry, and flow cytometry. An H_2_O_2_‐reduced human brain vascular smooth muscle cells (HBVSMC) injury model was also constructed.

**Results:**

The formation of aneurysms was observed in the circle of Willis in the IA mice. Melatonin treatment alleviated the thinning of blood vessel walls and disruption of the internal elastic lamina in IA mice. The levels of Bcl‐2 were significantly increased and Bax and cleaved caspase‐3 were decreased in IA mice with melatonin treatment, suggesting reduced apoptosis. Furthermore, melatonin reduced levels of interleukin (IL)‐1β, IL‐6, and tumor necrosis factor‐alpha (TNF‐α) in IA mice. The H_2_O_2_‐reduced HBVSMCs model showed consistent results. Melatonin reduced levels of krüppel‐like factor 6 (KLF6) in IA mice. Importantly, melatonin significantly reduced levels of regulatory T cells (Treg), hypoxia‐inducible factor (HIF)‐1α, nuclear factor interleukin 3‐regulated (NFIL3), TCDD‐inducible poly‐ADP‐ribose polymerase (TIPARP), and increased levels of monocytes in IA mice.

**Conclusion:**

Melatonin mitigates IA injury by modulating immune cells and hypoxia‐related factors. These findings provide an exploratory foundation for therapeutic strategies in IA.

## Graphical abstract

We established a mouse model of elastase‐induced intracranial aneurysms (IAs) to investigate the therapeutic potential of melatonin. Our findings suggest that melatonin may influence the formation and progression of IA by improving IA‐associated histological changes, regulating immune and inflammatory responses, and modulating the expression of hypoxia‐related genes. However, further studies are needed to optimize dosing, determine the ideal treatment timing, and assess specificity for clinical translation. Some images were obtained from SciDraw (https://doi.org/10.5281/zenodo.3925901, https://doi.org/10.5281/zenodo.3926245).

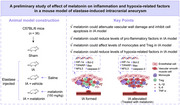

## 1 Introduction

Intracranial aneurysms (IAs) are present as pathologic dilatation of the walls of intracranial arteries, with a prevalence of 3% in the adult population (Etminan and Rinkel 2016). Although most patients with IA are asymptomatic, ruptured IAs can cause subarachnoid hemorrhage (SAH) with a high mortality rate (Claassen and Park 2022). The pathogenesis of IA involves multiple complex factors including hemodynamics, inflammation, and hypoxia (X. Wang et al. 2023). Changes in hemodynamics can induce an inflammatory response in endothelial cells, accumulation of immune cells, and the activation of pro‐inflammatory factors including interleukin (IL)‐1β, IL‐6, and tumor necrosis factor‐alpha (TNF‐α), which promote the formation of IA (Duan et al. 2023; Khan et al. 2023). Hypoxia can also induce the expression of pro‐inflammatory factors and is a key factor in IA formation and rupture (Ono et al. 2023).

Melatonin was first purified and characterized as an endogenous hormone, mainly secreted by the pineal gland, in 1958 (Lerner et al. 1958) and known to be involved in the regulation of sleep rhythms (Sletten et al. 2018) and antioxidant effects (Wakatsuki et al. 2001). Furthermore, melatonin has dual inflammatory and immunomodulatory effects, which remain controversial. Melatonin could participate in the early stages of inflammation by binding to phospholipase A2 (PLA2) via calmodulin, which immediately activates PLA2 and 5‐lipoxygenase (5‐LOX), resulting in the generation of pro‐inflammatory mediators (Radogna et al. 2009). Conversely, it also exerts appropriate anti‐inflammatory effects in experimental models of inflammatory and vascular diseases by inhibiting pro‐inflammatory factors (IL‐1β and TNF‐α), and genes (cyclooxygenase‐2 and inducible nitric oxide synthase) (Deng et al. 2006; Wang, Gao, et al. 2023). In addition, melatonin has shown potentially protective effects in animal models of various brain diseases (Perrone et al. 2023). For example, melatonin (10 mg/kg) may alleviate chronic cerebral underperfusion by modulating inflammation and brain barrier disruption (Thangwong et al. 2023). In clinical trials, melatonin has produced mixed results in its ability to control blood pressure, which may be linked to individual differences among participants (Amstrup and Rejnmark 2024; Cagnacci et al. 2005). Notably, melatonin's effects appear to be dose‐dependent, with different mechanisms and receptors potentially involved at varying concentrations (Oishi et al. 2021; Q. Wang et al. 2022). The role of melatonin in the diseases has not been fully investigated.

Our previous study revealed that melatonin was able to attenuate H_2_O_2_‐induced human brain vascular smooth muscle cell (HBVSMC) injury by downregulating Krüppel‐like factor 6 (KLF6) expression, and bioinformatics analysis for IA revealed that KLF6 was significantly correlated with immune cells such as monocytes, regulatory T cells (Treg), hypoxia‐related genes, hypoxia‐inducible factor (HIF)‐1α, nuclear factor interleukin 3‐regulated (NFIL3), and TCDD‐inducible poly‐ADP‐ribose polymerase (TIPARP) (Y. Liu et al. 2024). Based on these findings, melatonin may exert a therapeutic effect on IA by regulating these immune cells and hypoxia‐related genes. Therefore, we constructed a mouse model of IA to investigate the therapeutic effects of melatonin on IA and the involvement of the above immune cells and hypoxia genes in these effects.

## 2 Materials and Methods

### 2.1 Experimental Animal

Thirty‐six male C57BL/6 mice, aged 5–6 weeks, weighing 18–22 g, were purchased from SPF Biotechnology Co. Ltd (Beijing, China). Animal permit number: SCXK (E) 2020‐0018. All mice were kept in single cages under the following conditions: temperature of 24°C–26°C and humidity of 70%. The sample size was determined based on a power analysis using G*Power software (version 3.1). A minimum of eight mice per group (power = 80%, alpha = 0.05, Cohen's *f* = 0.25, medium effect size) was required for statistical validity. The mice were randomly divided into four groups (*n* = 9 per group): sham group, elastase was not injected; IA group, injected with elastase; IA + vehicle group, injected with elastase and treated with vehicle (saline); and IA + melatonin group, injected with elastase and treated with melatonin (dissolved in saline).

### 2.2 Animal Model

All mice were acclimatized for 7 days before the experiment. The elastase‐induced method was used to establish the IA model according to the previous studies (Abruzzo et al. 1998; Matsumoto et al. 2003). Briefly, mice were anesthetized with 5% isoflurane (RWD, China) for surgical procedures, including ligation of the renal pedicle and left carotid artery. After 1 week of rearing under normal conditions, the model groups (IA, IA + NC, and IA + melatonin) were intraperitoneally injected with elastase for cross‐pool stereotactic localization. After anesthetizing the mice, elastase was precisely administered into the basal cistern through the stereotaxic instrument (EZ Scientific, China), with the bregma serving as a landmark. The injection coordinates were set to 2.5 mm behind the bregma, 1.0 mm to the right, and 5 mm deep. A microinjection pump (Leadfluid, China), containing 5 µL (50 mU) of elastase (Yuanye, China) solution, was used to inject at a rate of 0.5 µL/min. After the operation, the mice were provided with 1% high‐saline water and standard food, with their health under close observation. Three days after surgery, 40,000 U penicillin was administered to prevent incision infection.

Melatonin has a low bioavailability, rapid metabolic rate, and short half‐life, characteristics that are more pronounced in rodents (Boutin et al. 2023; Choudhary et al. 2019). Therefore, the dose of 150 mg/kg was selected. In the IA + melatonin group, mice were injected intraperitoneally with melatonin (10 mg/mL, MCE, USA) at a dose of 150 mg/kg twice a week for 4 weeks. In the IA + vehicle group, mice were administered with an equal volume of saline. All other procedures remained consistent across both groups. To minimize bias, blinding was implemented during both the experimental procedures and data analysis.

### 2.3 Bromophenol Blue Assay

**FIGURE 1:**
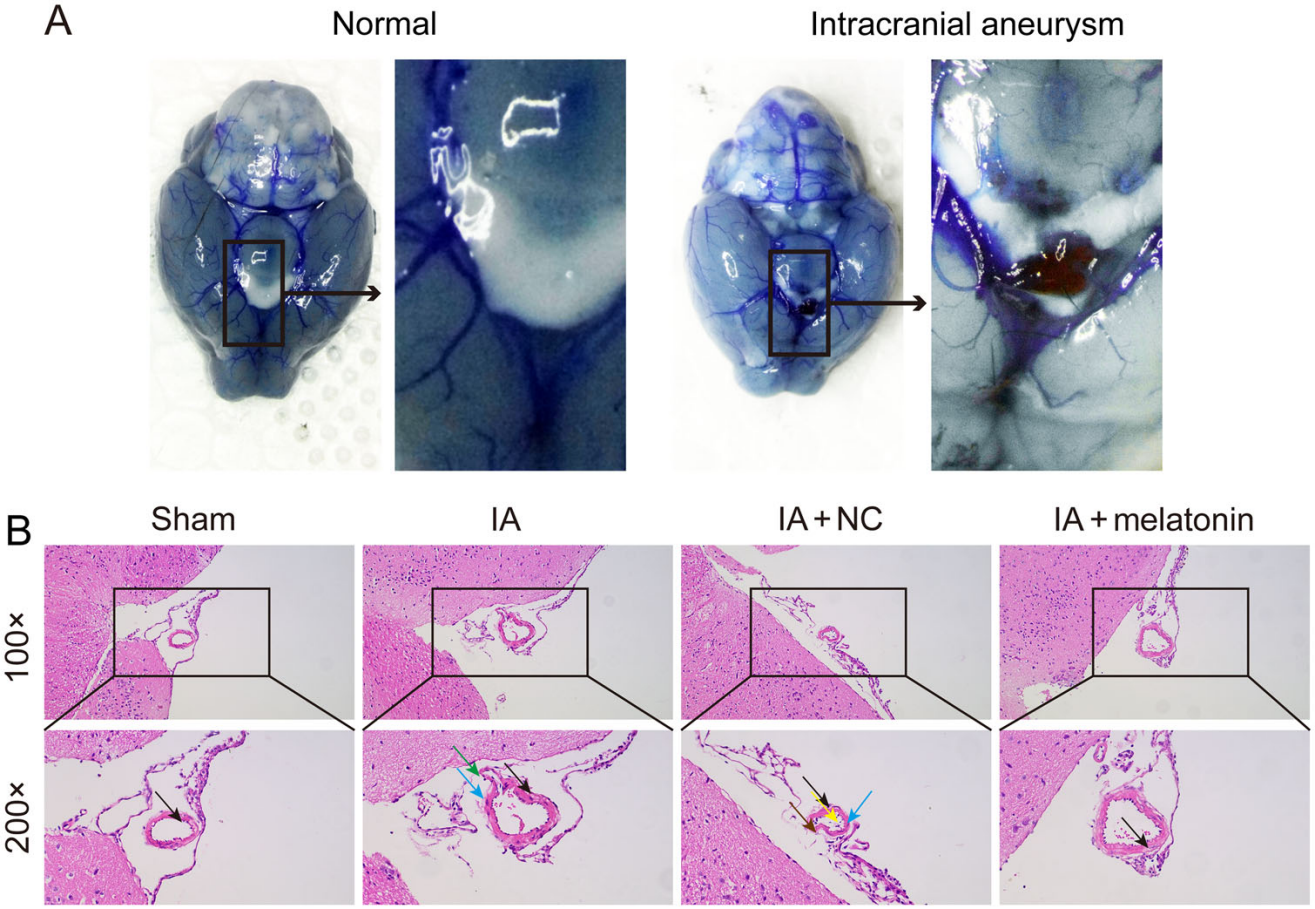
Melatonin suppresses vascular wall damage in IA mice. (A) Representative images of cerebral arteries. (B) Representative image of HE staining shows the destruction of vessel walls in the IA mouse model. *N* = 3. Cytoplasmic vacuolization (black arrows), disruption of the internal elastic lamina (blue arrows), infiltration of inflammatory cells (green arrows), endothelial cell detachment (yellow arrows), and nuclear condensation with hyperchromasia (brown arrows).

After 4 weeks, mice were anesthetized and the chest cavity was cut to expose the heart and collect blood. The right atrial appendage was cut and 3 mL of saline was perfused through the left ventricle. Subsequently, the procedure was repeated using bromophenol blue gelatin perfusion solution, containing 2 mg/mL bromophenol blue solution (Servicebio, China) and 20% gelatin. After incubating at 4°C for 5 min, the whole brain and cerebellum were removed from the mouse for observation under a stereomicroscope. To assess the tumorigenesis of IA, the diameter of the basilar artery was used as the reference criterion. If the diameter of the IA lesion vessel is dilated to a degree that exceeds 1.5 times the diameter of the reference basilar arterial vasculature, it is considered as an indication of IA tumorigenesis (Nuki et al. 2009). Representative aneurysms are shown in Figure 1. A subset of mice from each group (*n* = 3 per group) were perfused with saline, and the brain tissue was fixed in 4% paraformaldehyde for subsequent experiments.

### 2.4 Hematoxylin‐Eosin (HE) Staining

Mouse brain tissues were used to create paraffin‐embedded sections. The sections were dewaxed and rehydrated before staining with Mayer's hematoxylin (Keycell, China) for 5 min (clean staining background without differentiation). Following this, the sections were incubated with 1 x PBS for 5 min, and washed with tap water for 30 s. The sections were then stained with 1% water‐soluble eosin (Keycell) for 5 min and washed with tap water for 30 s. After dehydration, the sections were sealed and photographed. The basophilic structures were stained blue–purple and the acidophilic structures were stained pink.

### 2.5 Real‐Time qPCR (RT‐qPCR)

Total RNA was extracted from approximately half of the brain intracranial vascular tissues per mouse using TRIzol reagent (CWBIO, China) and reversed to cDNA using Evo M‐MLV (Accurate, China) reverse transcription reagent. The expression level of KLF6 was detected by RT‐qPCR using SYBR Green Pro Taq HS Mix (Accurate), with GAPDH serving as the internal reference gene. The reaction system was programmed as follows: incubation at 95°C for 30 s, followed by 40 cycles of 95°C for 5 s, and 60°C for 30 s. Relative expression was determined using the 2^−ΔΔct^ method. *p* < 0.05 was considered statistically significant. KLF6: forward primer: 5’‐GGCCCAAGTTTACCTCGACC‐3’; reverse primer: 5’‐TAGGCTTTCTCTCTCCCTGG‐3’; GAPDH: forward primer: 5’‐TTCTTTTGCGTCGCCAGGTG‐3’; reverse primer: 5’‐GGAGGGGAGAACAGTGAGC‐3’.

### 2.6 Immunohistochemistry (IHC)

Paraffin‐embedded sections were dewaxed and treated with 0.01 M Tris‐EDTA (CWBIO), boiling for 15 min. The sections were incubated with 3% H_2_O_2_ (CWBIO) for 15 min to block endogenous peroxidase. The sections were then blocked with goat serum for 30 min, followed by incubation with KLF6 antibody (rabbit, 1:200, Proteintech, China) at 4°C overnight. The sections were incubated with HRP‐labeled secondary antibody at 37°C for 30 min. The Sections were stained with DAB staining solution (Agilent, China) and Mayer's hematoxylin. Finally, the sections were dehydrated, sealed, and photographed. The sections were washed with 1 x TBS buffer between each step. For each group, three mice were analyzed, with three slides prepared per mouse, and three to four consecutive brain sections per slide. The percentage of positive cells was also analyzed by Aipathwell software, an AI‐based digital pathology image analysis tool that automatically locates and detects the target tissue on each slide. Positive cells were identified by brown or yellow–brown granules present in the cell membrane and cytoplasm, serving as positive indicators, while areas without staining granules were used as negative controls. In the Aipathwell software, the positivity criteria were set as brown or yellow–brown to enable the automatic labeling of positive cells and obtain the rate of positive cells.

### 2.7 Western Blot

Total protein was extracted from mouse brain intracranial vascular tissues using Enhanced RIPA lysis buffer, containing PMSF (BOSTER, China) and assessed by BCA Protein Assay Kit (Beyotime, China). Proteins were separated by 10% polyacrylamide gel (Servicebio) and electroblotted on PVDF membrane (Millipore, USA) through sodium dodecyl sulfate‐polyacrylamide gel electrophoresis (SDS‐PAGE). The membranes were blocked with 5% skimmed milk powder, followed by incubation with Bcl‐2 (1:2000, Abcam, China), Bax (1:20,000, Proteintech), cleaved caspase‐3 (1:1000, Abcam), NFIL3 (1:2000, Abcam), HIF‐1ɑ (1:5000, Abcam), TIPARP (1:500, Abcam), and GAPDH (1:50,000, Proteintech), at 4°C overnight. The membranes were incubated with HPR‐labeled secondary antibodies (1:5000). Finally, the protein band was visualized by ECL (Servicebio) and the densitometric analysis was performed by ImageJ (https://imagej.net/). The densitometric values of the target proteins were normalized to those of GAPDH, which was used as the internal control.

### 2.8 Enzyme‐Linked Immunosorbent Assay (ELISA)

The levels of IL‐1β, IL‐6, and TNF‐α in mouse serum were detected by IL‐1β kit (F2040‐B, Fanke Bio, Shanghai), IL‐6 kit (F2163‐B, Fanke Bio), and TNF‐α kit (F2132‐B, Fanke Bio) according to the manufacturer's instructions.

### 2.9 Flow Cytometry

The brain tissues of mice were ground and filtered using a strainer. The supernatant was discarded after centrifuging it at 4°C, 2000 r for 10 min. The Percoll density gradient centrifugation method was used to obtain leukocytes. Briefly, cells were resuspended with 7 mL of 1 x PBS and 3 mL of 100% Percoll. Next, 3 mL of 70% Percoll and 30% Percoll were added sequentially, and the mixture was centrifuged for 25 min at 4°C, 2000 r, with an acceleration of 3 and a deceleration of 2. The white cell layers were then aspirated and added to 1 x PBS and centrifuged for 10 min at 4°C, 2000 r, and this was repeated three times.

The single‐cell suspension was then divided into three groups: the blank group, the Monocytes group, and the Treg group. In the Treg group, cells in each tube were incubated with 1 µL of CD4‐FITC antibody (BioLegend, USA) in the dark for 30 min, and centrifuged at 1700 r/min for 7 min. The cells were then resuspended with 250 µL of Fix and Perm and incubated with FOXP3‐PE antibody (BioLegend) in the dark for 20 min. In the monocyte group, cells were incubated with 1 µL of CD14‐FITC (BioLegend) and PE anti‐mouse I‐A/I‐E antibodies (BioLegend). After washing with PBS, cells were assayed by a flow cytometer (NovoCyte, Agilent). Initially, a primary gate was set to select the main cell population (P1) based on forward scatter (FSC‐H) and side scatter (SSC‐H) to exclude debris and cell aggregates. Subsequently, monocytes were identified by gating on CD14 and HLA‐DR (I‐A/I‐E) positive signals, while Tregs were identified by gating on FOXP3 and CD4 positive signals.

The construction of the in vitro IA model using H_2_O_2_‐induced HBVSMCs and the corresponding experimental procedures are detailed in the Supporting Information.

### 2.10 Statistical Analysis

Data were statistically analyzed using SPSS 22.0 (IBMCorp, Armonk, N.Y., USA). The results were presented as mean ± standard deviation and visualized by GraphPad Prism 8 (www.graphpad.com, Boston, USA). Data are presented as the mean ± standard deviation. Normality and lognormality tests and Brown–Forsythe test were used to assess the normality and homogeneity of variance, respectively. The one‐way ANOVA was used to analyze sample comparisons between groups, followed by the Tukey multiple comparison tests. Fisher's exact test was used to compare the aneurysm formation rates. *p* < 0.05 was considered statistically significant.

## 3 Results

### 3.1 Melatonin Suppresses Vascular Wall Damage in IA Mouse Model

Approximately 13.8% (5/36) of the mice succumbed to intracranial hemorrhage and related complications. Four mice from each group were randomly selected for phenol blue gelatin infusion. The results showed that melatonin significantly reduced the formation of aneurysms in IA mice (sham vs. IA: 0% vs. 75%; IA + NC vs. IA + melatonin: 75% vs. 25%; Fisher's exact test, *p* = 0.038). We observed aneurysm formation using microscopy, and the results showed localized bulges in the circle of Willis in the IA mice (Figure 1A). Representative aneurysms and non‐aneurysms in each group are shown in Figure S1. The vascular morphology was observed by HE staining. Compared to the sham group, the IA mice exhibited thinning of the vascular walls, disruption of the internal elastic lamina, and focal lymphocytic infiltration around the vessels (Figure 1B). Compared to the IA + NC group, treatment with melatonin reduced vascular wall damage and exerted a protective effect against IA (Figure 1B).

### 3.2 Melatonin Inhibits Cell Apoptosis in IA Mouse Model

**FIGURE 2:**
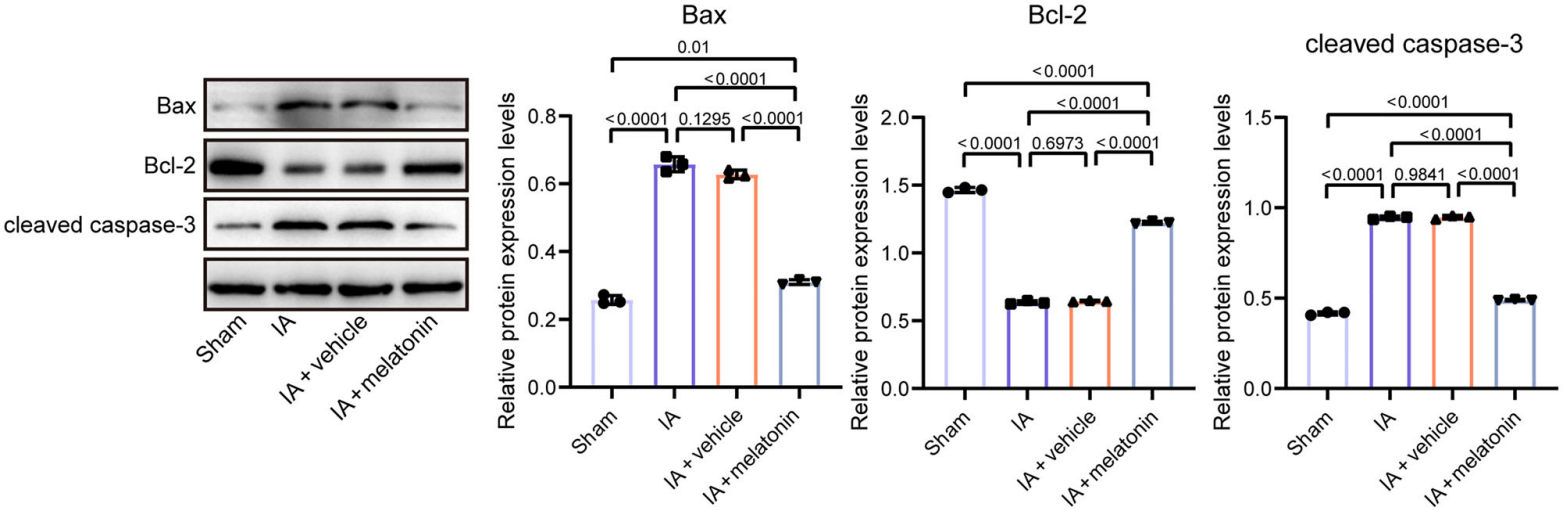
Melatonin inhibits cell apoptosis in IA mice. The levels of Bax, Bcl‐2, and cleaved caspase‐3 in mouse brain tissues were detected by western blot. Data represent three biological replicates and are presented as the mean ± SD.

Melatonin can regulate apoptosis through the mitochondrial pathway. In puromycin‐reduced U937 (human tumor monocytes) model, melatonin (1 mM) promotes the mitochondrial localization of Bcl‐2 and interferes with the activation of Bax, thereby inhibiting apoptosis and exerting its anti‐apoptotic effects (Radogna et al. 2008). We assessed the protein levels of the Bcl‐2 family. Compared to the sham group, the levels of the pro‐apoptotic factors Bax (*p* < 0.0001) and cleaved caspase‐3 (*p* < 0.0001) increased, while the level of the anti‐apoptotic factor Bcl‐2 (*p* < 0.0001) decreased in the IA group, suggesting enhanced apoptosis (Figure 2). Compared to the IA + NC group, apoptosis in the IA + melatonin group was significantly reduced (Figure 2).

### 3.3 Melatonin Reduces Pro‐Inflammatory Factor Levels in IA Mouse Model

**FIGURE 3:**
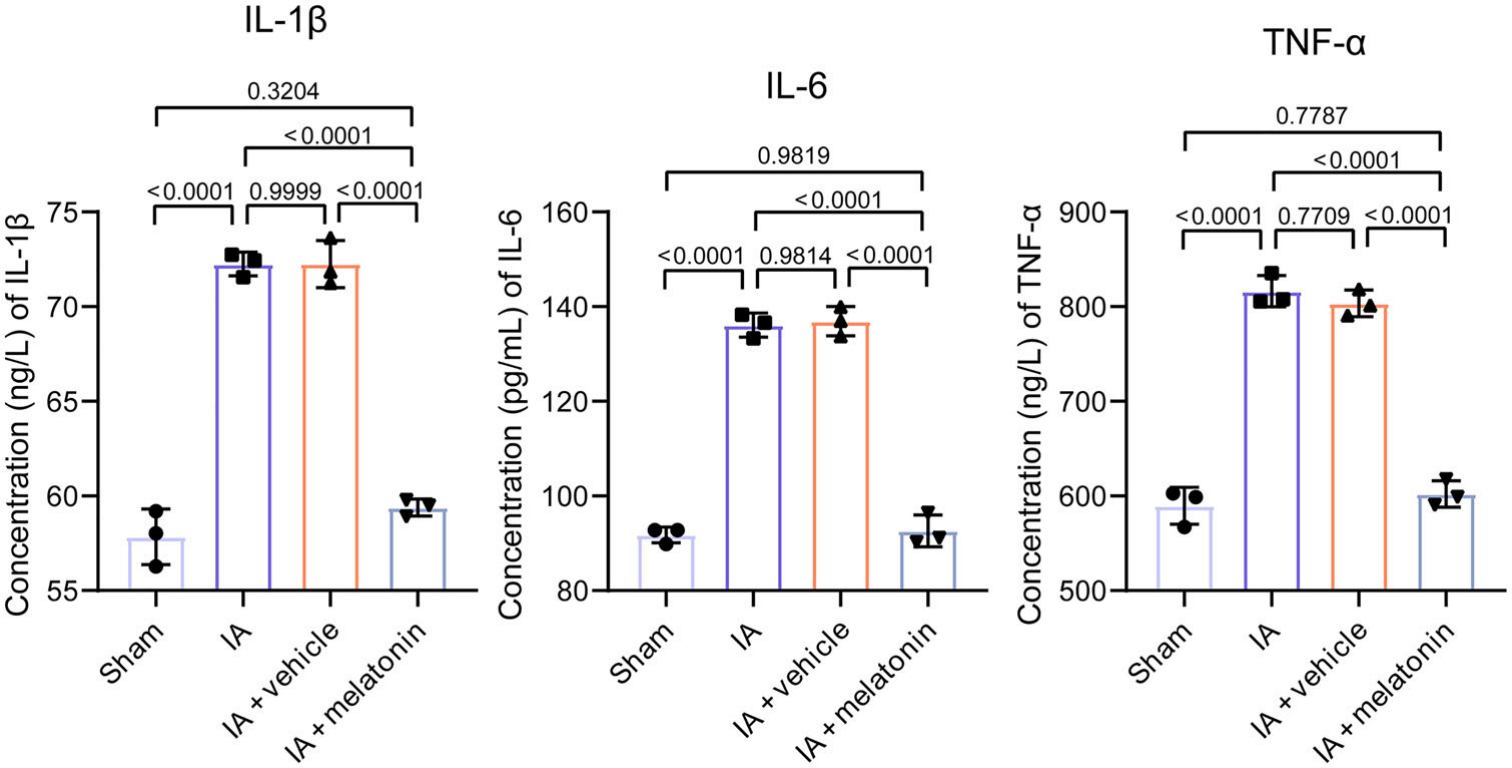
Melatonin reduces pro‐inflammatory factor levels. The levels of IL‐1β, IL‐6, and TNF‐α in mouse serum were detected by ELISA. Data represent three biological replicates and are presented as the mean ± SD.

IL‐1β, IL‐6, and TNF‐α are key pro‐inflammatory factors in the inflammatory process and play critical roles in the structural and functional remodeling of the vascular wall. ELISA results revealed that the levels of IL‐1β (*p* < 0.0001), IL‐6 (*p* < 0.0001), and TNF‐α (*p* < 0.0001) were significantly increased in the IA group compared to the sham group (Figure 3). Melatonin treatment reversed these results. As consistent with previous studies, we observed a decrease in proliferation and an increase in apoptosis of HBVSMCs after H₂O₂ treatment (Figure S2A–C), along with elevated levels of IL‐1β, IL‐6, and TNF‐α (Figure S2D), indicating aggravated injury. Meanwhile, melatonin was found to alleviate this injury (Figure S2).

### 3.4 Melatonin Affects Immune Cell Infiltration in IA Mouse Model

**FIGURE 4:**
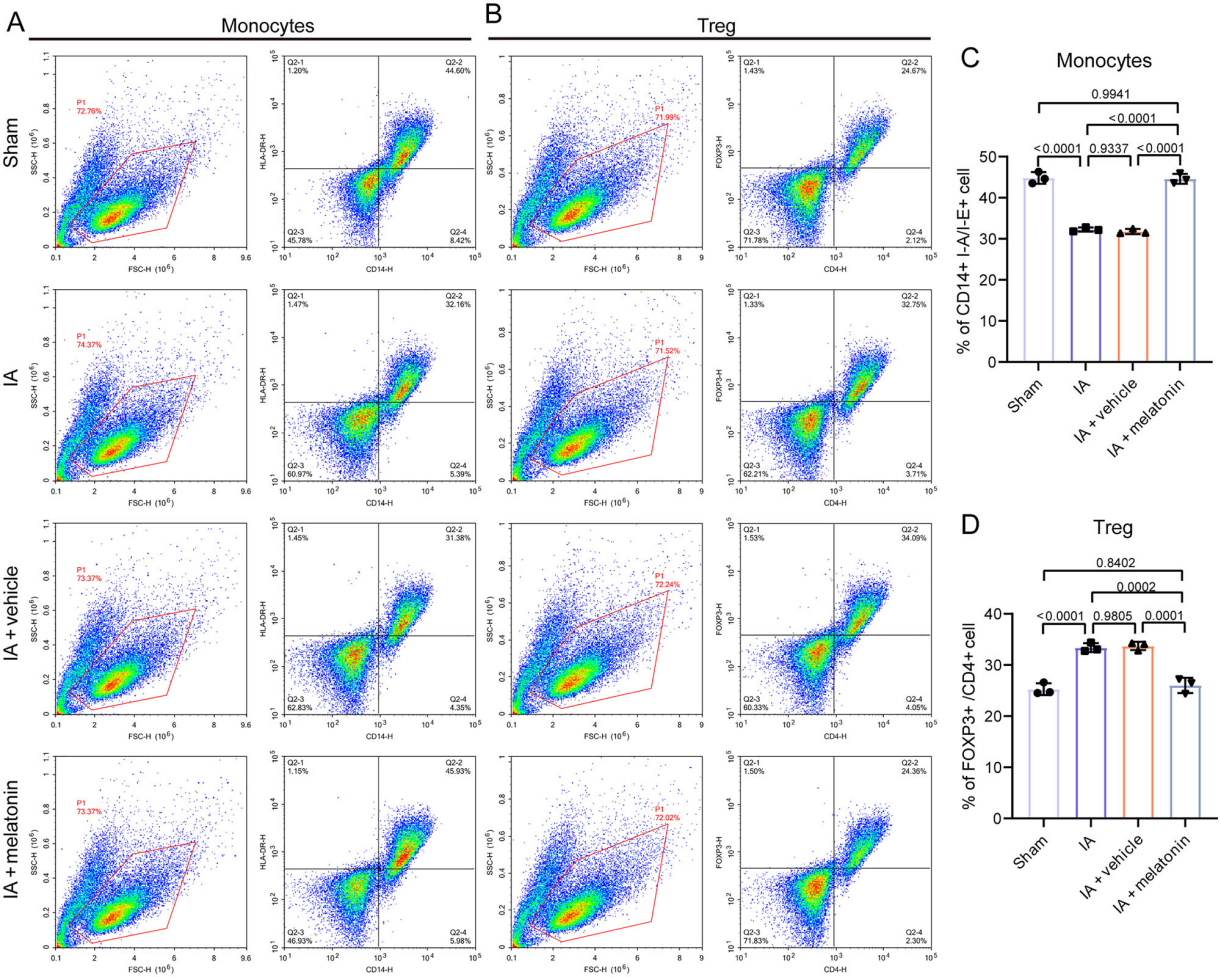
Melatonin affects immune cell infiltration. (A) The levels of monocytes (CD14^+^ I‐A/I‐E^+^ cells‐count number: sham group, 13,379; IA group, 9648; IA + vehicle, 9414; IA + melatonin group, 13,779) and Treg (CD4^+^ FOXP3^+^ cells‐count number: sham group, 7401; IA group, 9825; IA + vehicle, 10,227; IA + melatonin group, 7308) in mouse brain tissues were detected by flow cytometry and (B) presented as histograms. Bar graph comparing the percentage of (C) CD14^+^ HLA‐DR^+^ cells and (D) CD4^+^ FOXP3^+^ cells across the four groups. Data represent three biological replicates and are presented as the mean ± SD.

Compared to the sham group, the levels of monocytes were significantly reduced (*p* < 0.0001) and the levels of Treg were significantly increased (*p* < 0.0001) in the IA group (Figure 4). Compared to the IA + NC group, melatonin treatment significantly increased the levels of monocyte infiltration (*p* < 0.0001) and reduced the levels of Treg infiltration (*p* = 0.0001) in IA mice. In H₂O₂‐induced in vitro IA model, flow cytometry showed that the percentage of CD14^+^ HLA‐DR^+^ cells was decreased (*p* < 0.0001) and the percentage of CD4^+^ FOXP3^+^ cells was increased (*p* < 0.0001), suggesting the potential influence of monocyte and Treg pathways (Figure S3A–D). Compared to H₂O₂‐induced group, the melatonin group exhibited higher percentage of CD14^+^ HLA‐DR^+^ cells and lower percentage of CD4^+^ FOXP3^+^ cells (*p* < 0.0001).

### 3.5 Melatonin Reduces Levels of Hypoxia‐Related Factors in IA Mouse Model

**FIGURE 5:**
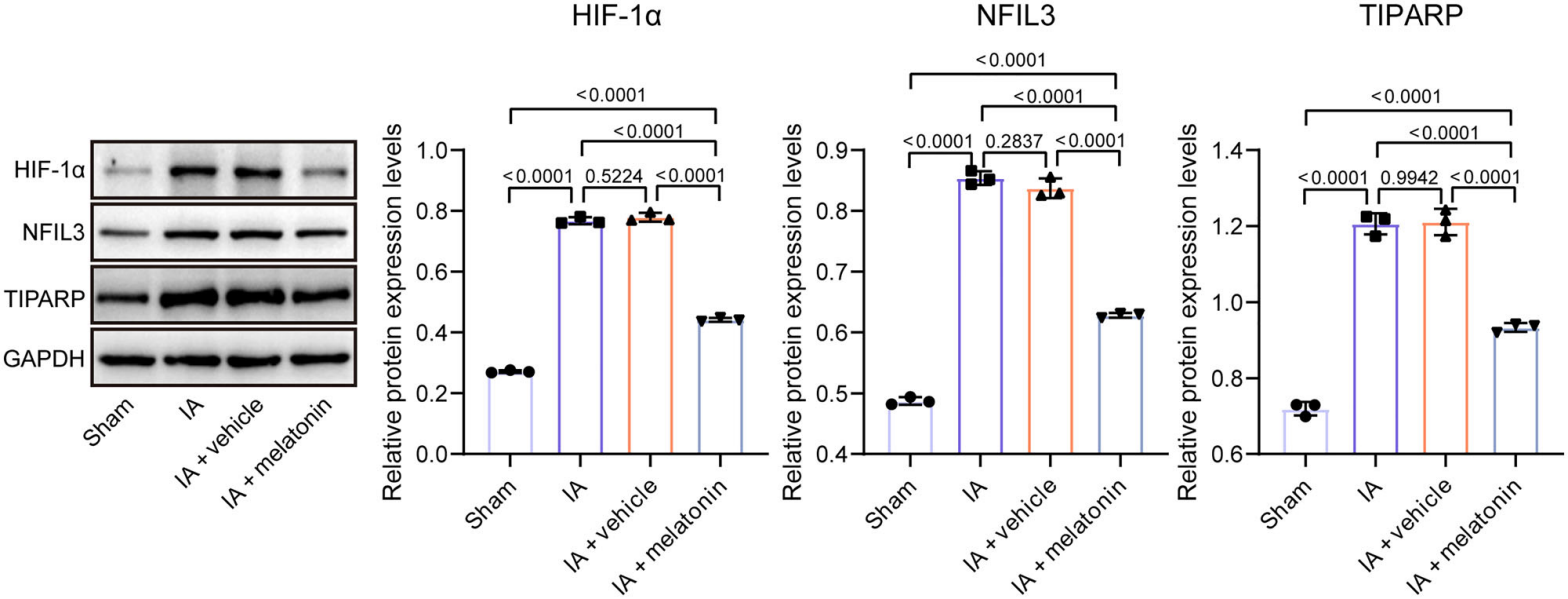
Melatonin reduces levels of hypoxia‐related factors. The levels of HIF‐α, NFIL3, and TIPARP in mouse brain tissues were detected by western blot and presented as histograms. Data represent three biological replicates and are presented as the mean ± SD.

The effects of melatonin on hypoxia‐related genes HIF‐1α, NFIL3, and TIPARP in the IA mouse model were investigated. Compared to the sham group, the levels of HIF‐1α (*p* < 0.0001), NFIL3 (*p* < 0.0001), and TIPARP (*p* < 0.0001) were significantly increased in the IA group (Figure 5). After melatonin treatment, the levels of HIF‐1α, NFIL3, and TIPARP were significantly decreased (*p* < 0.0001) in IA mice.

### 3.6 Melatonin Suppresses KLF6 Expression in IA Mouse Model

**FIGURE 6:**
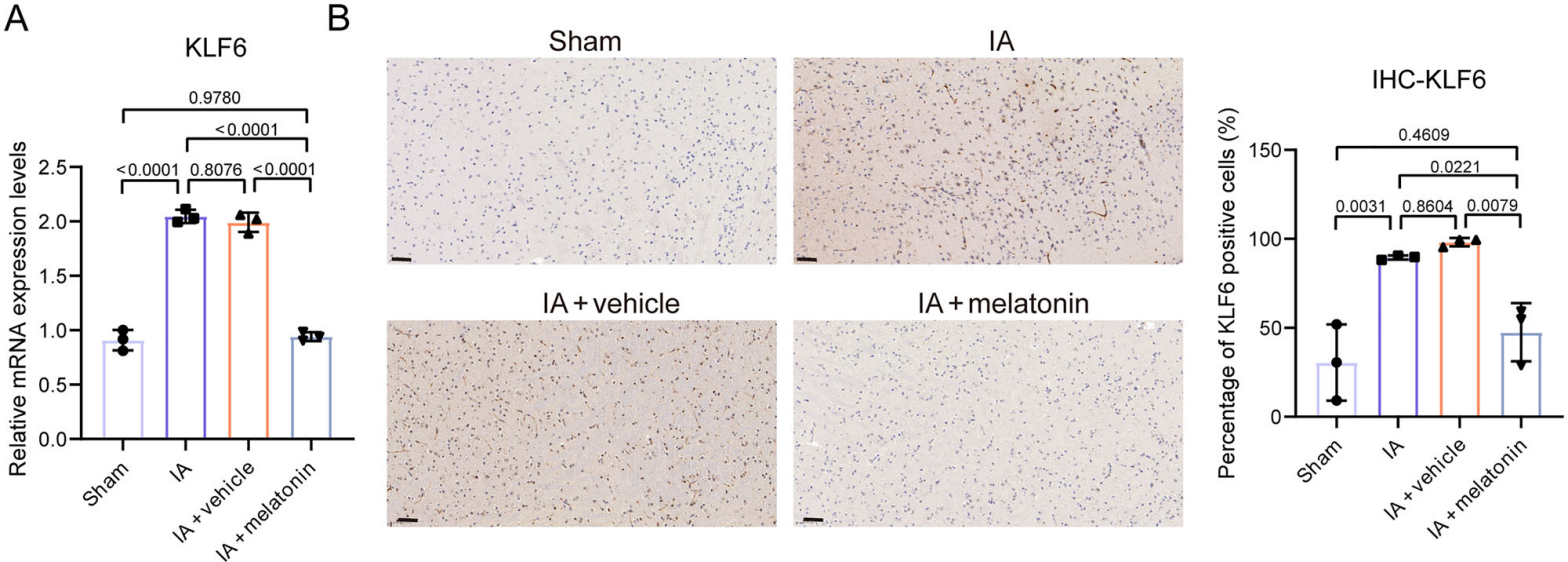
Melatonin suppresses KLF6 expression. (A) RT‐qPCR and (B) immunohistochemistry were used to assess KLF6 levels in mouse brain tissues. Scale bars = 50 µm. Data represent three biological replicates and are presented as the mean ± SD.

RT‐qPCR and IHC were used to detect the levels of KLF6. Compared to the sham group, the levels of KLF6 were significantly increased (*p* = 0.0031) in the IA group (Figure 6). After melatonin treatment, the levels of KLF6 were decreased (*p* = 0.0079) in IA mice.

## 4 Discussion

IA is the leading cause of non‐invasive SAH and poses a huge burden on global health (Kane et al. 2023). The current treatment options for IA, such as aneurysm clamping and endovascular interventions, are invasive and can lead to various complications (Deshmukh et al. 2024). Therefore, it is important to find effective and safe treatment options. This study is the first to investigate the therapeutic effect of melatonin on IA and its possible mechanisms through an in vivo model, providing new insights into the treatment of IA.

It has been shown that patients with normal pressure hydrocephalus caused by IA rupture lack a natural melatonin rhythm (Yamada et al. 1991). Furthermore, there was a significant correlation between serum melatonin levels and functional outcomes in SAH (Zhan et al. 2021). Previous studies have demonstrated the potential benefit of melatonin in the treatment of SAH. For example, melatonin could significantly reduce mortality, decrease brain edema, and maintain the integrity of the blood–brain barrier in SAH rats (Ayer et al. 2008; Ersahin et al. 2009). In addition, severe histologic‐morphologic changes were observed in IA, as well as disturbances in VSMC cell proliferation and apoptosis, which greatly influence the formation and progression of IA (Wang, Ma, et al. 2023). Our study demonstrated that melatonin treatment alleviated the thinning of blood vessel walls, disruption of the internal elastic lamina, and reduced cell apoptosis in IA mice. These findings suggested that melatonin may offer new therapeutic possibilities for IA and deserves further investigation.

It is crucial to understand the mechanisms of melatonin treatment for IA. Levels of inflammatory and pro‐inflammatory factors are strongly associated with the formation and risk of IA (Duan, et al. 2023; H. F. Zhang et al. 2013). Our study showed significant tissue inflammation and upregulation of inflammatory markers IL‐1β, IL‐6, and TNF‐α in IA mice, consistent with previous research (Shimada et al. 2015). Melatonin treatment reversed the upregulation of these inflammatory markers. Furthermore, consistent results were observed in the H_2_O_2_‐induced HBVSMC injury model. We also observed an increase in the levels of monocyte infiltration after melatonin treatment, which may suggest that melatonin promotes the transformation of monocytes to an anti‐inflammatory phenotype, aiding in the repair and stabilization of IA (Muhammad et al. 2021). Moreover, the levels of Treg infiltration decreased after melatonin treatment. Treg plays an important role in maintaining immune homeostasis (Li et al. 2024). Therefore, melatonin may create a more favorable immunoregulatory environment in IA mice by affecting the expression of pro‐inflammatory factors and the infiltration of monocytes and Treg. However, it is important to note that the effects of melatonin may be highly context‐dependent. Studies have shown that melatonin can stimulate the production of monocytes in the bone marrow and spleen of mice (Currier et al. 2000) and modulate their cytokine secretion capacity (Morrey et al. 1994), with these effects being concentration‐dependent. Moreover, recent studies indicate that at pharmacological levels (≥ 1 µM), melatonin does not appear to enhance immune‐inflammatory responses (Boutin et al. 2024). This may be attributed to receptor desensitization and internalization, which are thought to result in putative non‐receptor‐mediated effects. Given the pleiotropic nature of melatonin, its functions should be interpreted with caution.

The hypoxic environment is a key factor in IA progression, which could promote angiogenesis and inflammatory cell infiltration in IA (Ono et al. 2023). Studies have shown that HIF‐1α upregulation plays a role in IA progression by modulating VSMC phenotype and macrophage polarization toward M1 (Gao et al. 2019; Han et al. 2023). Under hypoxic conditions, HIF‐1α also leads to the activation of TIPARP and NFIL3, which affect the antioxidant capacity and immune status of cells (Douanne et al. 2024; Gozgit et al. 2021; Yu et al. 2024; L. Zhang et al. 2020). Boutin and Jockers (2021) reviewed melatonin's role in oxidative stress and proposed that melatonin acts as an inducer of cellular antioxidant defenses rather than as a scavenger of ROS. Consistent with this, our study showed that melatonin treatment could reduce the levels of HIF‐1α, TIPARP, and NFIL3 in IA mice, further suggesting that melatonin may treat IA by attenuating hypoxia‐induced cellular stress and inflammatory responses through enhancing antioxidant defense mechanisms.

KLF6 belongs to the family of zinc finger DNA‐binding proteins that regulate various functions of cells (Syafruddin et al. 2020). It plays a role in regulating macrophage polarization and pro‐inflammatory factor expression (Date et al. 2014) and can initiate the hypoxic response by regulating HIF‐1α expression in macrophages (Kim et al. 2020). Previous research has shown that KLF6 can bind to p300 and p300/CBP‐related factors to activate transcription (Yuce and Ozkan 2024). Furthermore, melatonin can inhibit p300 and regulate apoptotic pathways in cancer cells (J. Wang et al. 2012), indicating that melatonin may influence KLF6 activity. In addition, KLF6 promotes IA progression in an in vitro model (Yue et al. 2022). Our study showed that melatonin reduced KLF6 expression in IA mice, providing further support for the previous study.

Our findings highlight the effects of melatonin on vascular wall damage, inflammation, and immune modulation in IA, offering additional therapeutic possibilities. However, the pleiotropic nature of melatonin may limit its specificity as a therapeutic agent (Boutin et al. 2023). The doses used in this study were selected based on their established efficacy in animal models (Chen et al. 2015; Hou et al. 2020), but their high levels pose challenges for clinical translation. Previous clinical studies have utilized high doses of melatonin, including 3 g/day for 6 months (with no reported safety issues) (D'Anna et al. 2017), 1 g/day for 1 month (no adverse effects) (Nordlund and Lerner 1977), and 50 mg/kg (well‐tolerated and safe) (Nickkholgh et al. 2011). However, exogenous melatonin also has some moderate to mild adverse events (Besag et al. 2019). Therefore, the safety of high‐dose melatonin in humans has not been thoroughly validated. Furthermore, oral melatonin (0.3–100 mg) has a low bioavailability, ranging from 9% to 33% (Harpsøe et al. 2015), which may suggest the potential benefit of exploring higher doses of melatonin to achieve more effective therapeutic outcomes. Nevertheless, high doses of melatonin could result in transiently elevated plasma concentrations, potentially reaching millimolar levels, which may lead to non‐specific interactions with a broad range of proteins and enzymes (L. Liu et al. 2019). These considerations emphasize the need for cautious interpretation of preclinical findings at high doses and highlight the importance of further studies to optimize dosing strategies and minimize off‐target effects.

This study has several limitations. First, the sample size is limited, which may potentially reduce the statistical power of our findings. Second, high dose of melatonin poses challenges for clinical translation. Future studies should focus on identifying the specific pathways involved using larger sample size and determining the optimal effective dose to bridge the gap between preclinical and clinical research. Third, this study did not include a sham‐operated group to assess the effects of melatonin alone. Finally, although modifications to the 5‐methoxy and acylamino groups, side chain positions, and lipophilic/hydrophilic balance of indole derivatives can significantly influence their antioxidant and cytoprotective activities (Mor et al. 2004), the differing effects of melatonin and other indole‐based compounds at high doses remain unclear. Future research should focus on comparing their efficacy to better understand the specific role of melatonin in the pathology of IA.

## 5 Conclusion

In conclusion, melatonin affects the formation and progression of IA by ameliorating IA‐associated histologic changes, modulating immune and inflammatory responses, and the expression of hypoxia genes and KLF6. These findings suggest that melatonin may provide new therapeutic possibilities for IA, providing a basis for further studies. However, its clinical translation requires additional investigation to optimize dosing, assess treatment timing, and evaluate specificity.

## Author Contributions

**NarenYa**: writing–original draft, data curation, conceptualization, methodology, investigation, resources. **Yan Feng**: conceptualization, validation, visualization, data curation, resources, writing–original draft. **Yongxing Su**: data curation, investigation, resources, methodology. **Le Chen**: data curation, resources, validation. **Yan Liu**: resources, data curation, validation. **Zhongwu Sun**: resources; data curation, writing–review and editing. **Zhengfei Ma**: resources, data curation, conceptualization, writing–review and editing.

## Ethics Statement

The approval of the research was obtained through the Animal Ethics Committee of the Anhui Medical University (LLSC20241005). All methods were conducted in accordance with the ARRIVE guidelines (https://arriveguidelines.org).

## Conflicts of Interest

The authors declare no conflicts of interest.

### Peer Review

The peer review history for this article is available at https://publons.com/publon/10.1002/brb3.70371.

## Supporting information



Supporting Information

## Data Availability

The data that support the findings of this study are available from the corresponding author upon reasonable request.
