# Supplementary material for: A Preliminary Study of Effect of Melatonin on Inflammation and Hypoxia‐Related Factors in a Mouse Model of Elastase‐Induced Intracranial Aneurysm
Source: Brain Behav. 2025 Feb 28;15(3):e70371. doi: 10.1002/brb3.70371 (PMC11870834; doi:10.1002/brb3.70371)
Supplement: Supplementary file 1 — Supporting Information [file BRB3-15-e70371-s001.docx]

**Supplementary materials and methods**

**Cell culture and protocols**

HBVSMCs (Cat. No. CM-H116) were obtained from Procell (Wuhan, China) and maintained in DMEM supplemented with 10% fetal bovine serum (FBS) at 37°C in a 5% CO2 atmosphere. Cells digested with trypsin were seeded into 6-well plates at a density of 2×10⁵ cells/well. The adherent cells were divided into three experimental groups: control, H_2_O_2_, and H_2_O_2_+melatonin. Both the H_2_O_2_ and H_2_O_2_+melatonin groups were treated with 100 μM H_2_O_2_ for 6 hours, while the control group received an equivalent volume of PBS. In the H_2_O_2_+melatonin group, 100 μM melatonin was additionally administered for 6 hours after H_2_O_2_ treatment.

**Cell apoptosis assay**

Apoptosis was assessed using the Annexin V-PE/7-AAD Apoptosis Assay Kit (Meilunebio, China) following the manufacturer’s protocol. Three control conditions were established: normal cells, cells stained with 7-AAD, and cells stained with Annexin V-PE. The analysis was performed using CellQuest software, generating two-color dot plots with PE on the x-axis and 7-AAD on the y-axis. Apoptosis was quantified as the sum of Annexin V-PE^+^ 7-AAD^+^ and Annexin V-PE^+^ cell percentages.

**3-(4,5-dimethylthiazol-2-yl)-2,5-diphenyltetrazolium bromide (MTT) assay**

For the MTT assay, cells were seeded into 96-well plates at a density of 6000 cells per well and cultured for 24, 48, 72, and 96 h. At each time point, 20 μL of 5 mg/mL MTT solution (Sangon Biotech, China) was added to the wells and incubated for 4 h. The culture medium was then completely removed, and 100 μL DMSO was added to dissolve the formazan crystals. The optical density (OD) at 490 nm was measured using a microplate reader (Thermo Fisher, USA). The fold increase in proliferation for each group at 48, 72, and 96 h was calculated relative to the OD value at 24 h.

**ELISA**

The levels of IL-1β, IL-6, and TNF-α in the cell supernatant were detected by IL-1β kit (F0179-B, Fanke Bio, Shanghai), IL-6 kit (F0049-B, Fanke Bio), and TNF-α kit (F0121-B, Fanke Bio) according to the manufacturer’s instructions.

**Immune cell marker assay**

The cell pellets collected after trypsin digestion were washed with pre-cooled PBS. After centrifugation at 1500 rpm for 5 minutes, the supernatant was discarded. For the detection of monocyte-associated markers, 1 μL of fluorescently labeled CD14 antibody (BioLegend, Cat#301847) and HLA-DR antibody (BioLegend, Cat#327007) were added to the cells and incubated in the dark at room temperature for 30 minutes. Following incubation, the cells were washed with PBS and resuspended in 500 μL PBS for flow cytometry analysis using a NovoCyte instrument. For the detection of Treg-associated markers, 1 μL of fluorescently labeled CD4 antibody (BioLegend, Cat#317431) was added to the cells and incubated in the dark at room temperature for 30 min. After discarding the supernatant, the cells were fixed and permeabilized by adding 250 μL of Fix and Perm reagent, followed by incubation in the dark at room temperature for 20 min. The cells were then washed with 1x buffer and incubated with 1 μL of fluorescently labeled FOXP3 antibody (BioLegend, Cat#320207) in the dark for 60 minutes. After a final wash with 1x buffer, the cells were resuspended in 500 μL of 1x buffer and analyzed by flow cytometry using a NovoCyte instrument.

**Supplementary Figures**

**
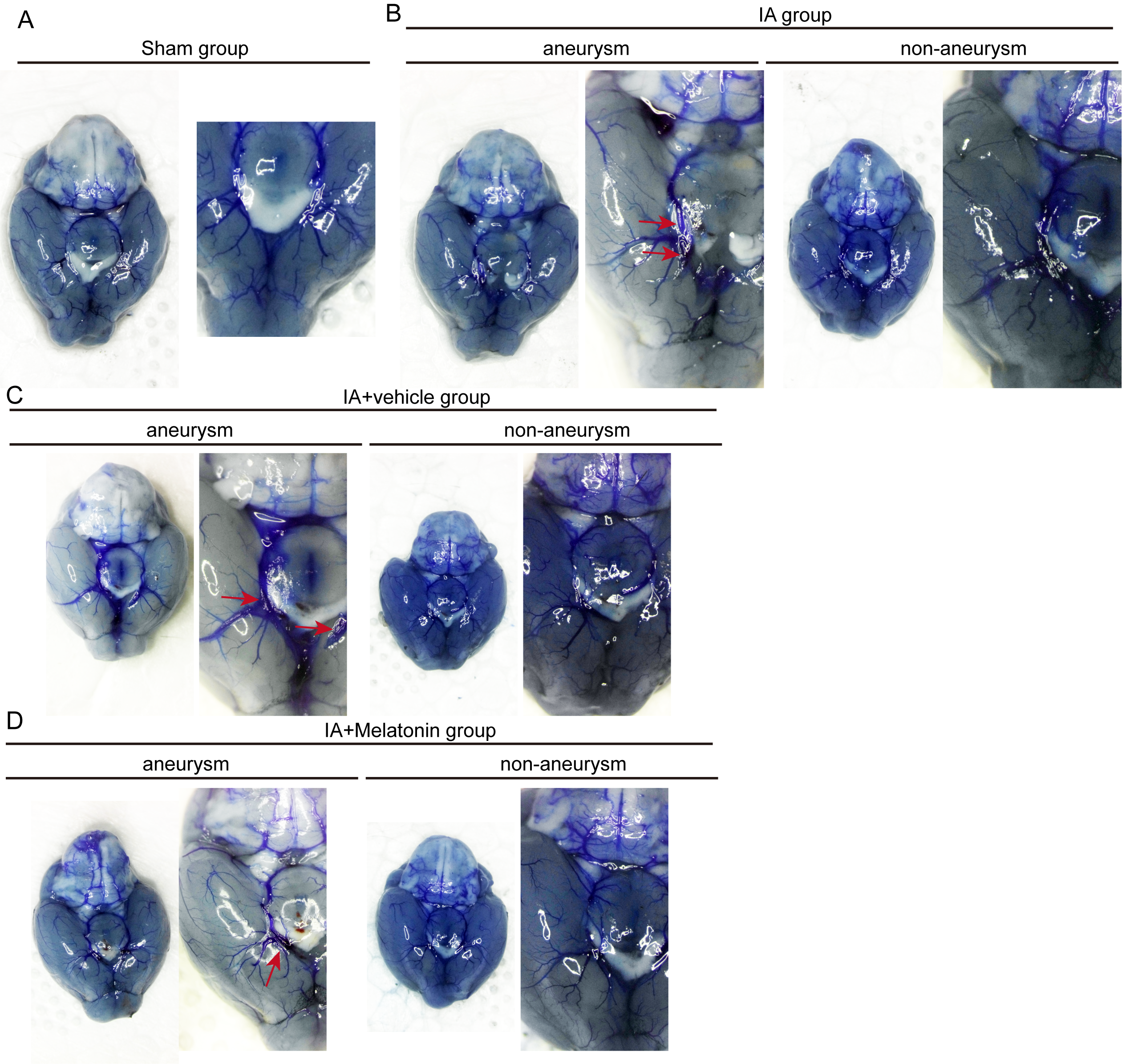
**

**Figure S1. Observation of aneurysm formation in mice**

Representative aneurysms or non-aneurysms in the (A) sham, (B) IA, (C) IA+vehicle and (D) IA+melatonin group. Red arrows indicate the location of aneurysms.


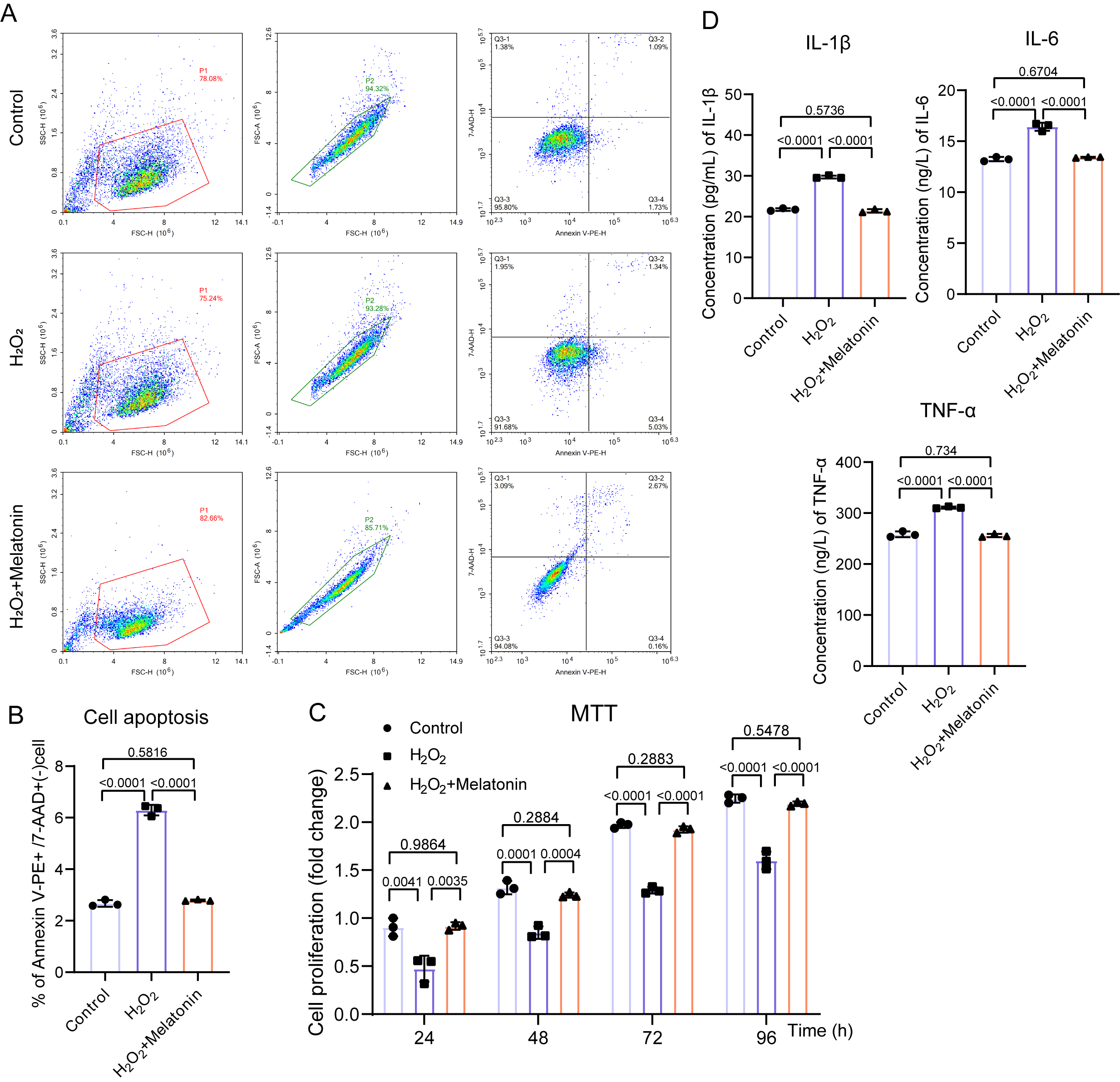


**Figure S2. Melatonin reduces pro-inflammatory factor levels in the H_2_O_2_-induced HBSMC injury model**

(A) Flow cytometry was performed to detect HBVSMC cell apoptosis. (B) Apoptosis was quantified as the sum of Annexin V-PE^+^ 7-AAD^+^ and Annexin V-PE^+^ cell percentages. (C) MTT was performed to detect HBVSMC cell proliferation. (D) ELISA was used to evaluate the levels of IL-1β, IL-6, and TNF-α in cell supernatant. Data represent three biological replicates and are presented as the mean ± SD.


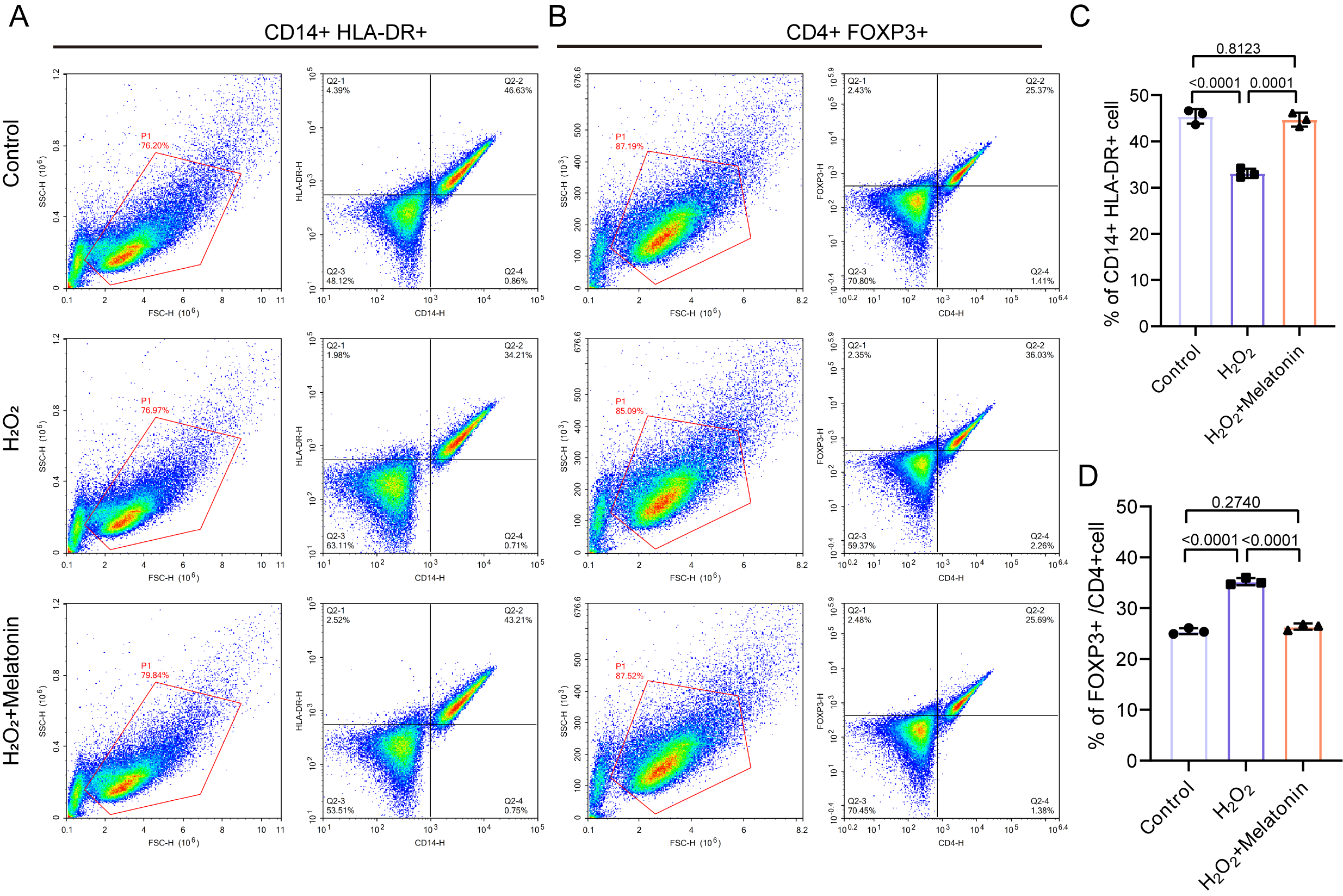


**Figure S3. Melatonin reduces pro-inflammatory factor levels in the H_2_O_2_-induced HBSMC injury model**

Flow cytometry was performed to detect the percentage of (A) CD14^+^ HLA-DR^+^ cells (count number: control group, 23285; H_2_O_2_ group, 17104; H_2_O_2_+melatonin group, 20347) and (B) CD4^+^ FOXP3^+^ cells (count number: control group, 12684; H_2_O_2_ group, 18016; H_2_O_2_+melatonin group, 12844). Bar graph comparing the percentage of (C) CD14^+^ HLA-DR^+^ cells and (D) CD4^+^ FOXP3^+^ cells across the three groups. Data represent three biological replicates and are presented as the mean ± SD.
